# Elderly Medication Adherence Intervention Using the My Interventional Drug-Eluting Stent Educational App: Multisite Randomized Feasibility Trial

**DOI:** 10.2196/15900

**Published:** 2020-06-24

**Authors:** Andrew Dallas Boyd, Chioma Iheanyi Ndukwe, Anandu Dileep, Olivia Frances Everin, Yingwei Yao, Betty Welland, Jerry Field, Matt Baumann, Jose D Flores Jr, Adhir Shroff, Vicki Groo, Carolyn Dickens, Rami Doukky, Regeena Francis, Geraldine Peacock, Diana J Wilkie

**Affiliations:** 1 Department of Biomedical and Health Information Science University of Illinois at Chicago Chicago United States; 2 Biobehavioral Nursing Science University of Florida Gainesville, FL United States; 3 Patient Advisor, Department of Biomedical and Health Information Sciences University of Illinois at Chicago Chicago, IL United States; 4 Department of Pharmacy Practice University of Illinois at Chicago Chicago, IL United States; 5 Divison of Cardiology Cook County Health Chicago, IL United States

**Keywords:** mobile application, medication adherence, drug eluting stents, percutaneous coronary intervention, patient education, Kolb learning theory

## Abstract

**Background:**

A lifesaving treatment for myocardial infarction is the placement of a stent in a closed or obstructed coronary artery. The largest modifiable risk factor after receiving a stent is medication adherence to Dual AntiPlatelet Therapy, a combination of P2Y12 inhibitors and aspirin.

**Objective:**

This study aimed to determine the acceptability of a protocol and an intervention using the My Interventional Drug-Eluting Stent Educational App (MyIDEA) and to evaluate medication adherence using the proportion of days covered (PDC) and platelet activation tests in a multisite randomized controlled trial.

**Methods:**

Potential participants who received a post percutaneous coronary intervention (PCI) procedure with a drug-eluting stent were approached. All patients older than 50 years and who spoke English were recruited. Participants were recruited, baseline demographics were collected, and the Hospital Anxiety and Depression Scale (HADS), Rapid Estimate of Adult Literacy in Medicine-Short Form, Burden-Benefit questionnaire, 36-Item Short Form Health Survey, and PCI knowledge questionnaire were administered. Block randomization was used to randomize participants to either usual care or MyIDEA supplementation. MyIDEA is a personalized educational intervention based on the Kolb experiential learning theory using patient narratives for education. During the visits, participants’ blood was collected to measure platelet suppression from medication. During the second and third encounters, the Morisky medication adherence score and cardiology outcomes were measured. The study was conducted at the University of Illinois Hospital and John H Stroger Jr Cook County Hospital with appropriate ethical approvals. Platelet suppression was measured through aspirin reactive units and P2Y12 reactive units. Medication adherence was measured using the PDC. The analysis team was blinded to the participants’ group membership. The primary outcome was a feasibility analysis of recruitment and retention.

**Results:**

The mean age of participants was 60.4 years (SD 7.1); the majority of patients were black and non-Hispanic. The majority of patients’ reading levels were seventh grade or above, and they were not very familiar with other electronic devices for information and communication. The number of control subjects was 21, and the number of participants in the interventional arm was 24. The interventional group was able to use MyIDEA in both the hospital and outpatient setting. However, there was no significant difference in platelet suppression or medication adherence between groups. There were also differences between the groups in terms of depression and anxiety, initially, as measured by HADS. No documented adverse event associated with the intervention was found.

**Conclusions:**

Elderly patients are willing to use tablet devices to be educated about health conditions. Additional studies are required to measure the effectiveness and determine the most suitable timing and location for patient education.

**Trial Registration:**

ClinicalTrials.gov NCT04439864; https://clinicaltrials.gov/ct2/show/NCT04439864

## Introduction

### Background

A lifesaving treatment for myocardial infarction is the placement of a stent in a closed or an obstructed coronary artery [1]. The placement of a stent restores blood circulation to areas of the heart for patients with coronary artery disease. The most commonly used stent in the United States is the drug-eluting stent (DES) [2]. In addition, the largest modifiable risk factor after receiving a stent is medication adherence to dual antiplatelet therapy (DAPT), a combination of P2Y12 inhibitors and aspirin [3]. By stopping the medication, the risk of death is increased seven times compared with patients who follow medication directions [4-7]. The range of medication nonadherence has been reported from 7% to 50% [8,9]. Even missing only 48 hours of medication has been reported to increase the risk of stent thrombosis [10]. Current literature has shown that 5.4% of patients do not pick up their medication from the pharmacy [3]. The most common reasons for stopping their medication were a lack of understanding and not knowing how long they would need to take the medication [11-13].

A review of a number of different methods have been attempted to increase medication adherence in patients poststent [14]. A pharmacy-led intervention [15] has not been shown to increase medication adherence. Another challenge is that a lower socioeconomic status has been affiliated with lower health-seeking behavior [16]. One large area of innovation is in engaging patients in mobile health. Although currently there are over 100,000 apps to download from the Google Play and Apple App stores, very few have been studied in randomized controlled trials [17], and the apps are written at a grade level higher than the reading level of the patients [18]. The strength of using mobile health technology lies in its ability to engage the patient via customized interventions [19,20].

The My Interventional DES Educational App (MyIDEA) was a computing tablet-based education program developed with patients, cardiologists, pharmacists, nurses, informaticians, and biomedical illustrators [21]. MyIDEA was developed using the Kolb experiential learning theory such that it was customized to the patient [22]. MyIDEA also used patient narratives to help the patient plan and overcome the common barriers to medication adherence.

### Objectives

This study aimed to determine the acceptability and the effect of MyIDEA medication adherence through proportion of days covered (PDC) and platelet activation tests in a multisite randomized controlled trial. MyIDEA offers an immediate response to common questions after stent procedures when the patient is at home and reviews medical instructions from nursing staff.

## Methods

A feasibility study was conducted at 2 urban hospitals, the University of Illinois Hospital and John H Stroger Jr Cook County Hospital, to evaluate the medication adherence of patients availing DAPT. Both hospitals care for patients with a low socioeconomic status and have a high percentage of minority patients. The participants were recruited from University of Illinois Hospital from May 2014 to February 2016 and from John H Stroger Jr Cook County Hospital from June 2016 to February 2017.

### Recruitment

The study was approved by the institutional review boards of both institutions. The enrollment criteria included patients who (1) received a DES in a percutaneous coronary intervention (PCI) during a hospitalization for the PCI procedure, (2) were older than 50 years (funding agency requirement), and (3) spoke and understood English. The exclusion criteria were as follows: (1) inability to give informed consent and (2) allergy to aspirin. The change to the study design was to include a second site.

The participants were approached in person for recruitment between 4 and 24 hours after their PCI procedure and before being discharged home. The enrollment delay was designed to allow patient recovery from the procedure that would allow for sufficient awareness after the procedure and reduce potential effects of sedation on the readiness to learn. As the study was a pilot, and owing to limited funds, a power analysis was not conducted. After enrollment, a study coordinator collected baseline demographic details. The age and gender of each of the participants were recorded. The races of the participants were white, black/African American, American Indian/Alaska Native, Asian, or multiracial. For ethnicity, the participants were either Hispanic or non-Hispanic. Participants were also asked about their social support network and if they lived with family or had friends that could pick up medication for them, and the percentage of participants with assistance was determined.

The following psychometric evaluations were administered: Hospital Anxiety and Depression Scale (HADS) [23], Rapid Estimate of Adult Literacy in Medicine-Short Form (REALM-SF) [24], Burden-Benefit questionnaire [25], 36-Item Short Form Health Survey (SF-36) [26], and PCI knowledge questionnaire [27] (Multimedia Appendix 1). The REALM-SF scored literacy using grade levels from below a third-grade reading level to a high school reading level and above. HADS scored anxiety and depression by summing the values of the anxiety and depression answers. The SF-36 scores were divided into 6 sections—physical functioning, role limitations because of physical health and because of emotional problems, energy and fatigue, emotional well-being, social functioning, pain, and general health—and the scores were determined by summing weighted values from the answers to the questionnaire and then taking the average of those values for each subscore. The PCI knowledge questionnaire scored participants on the accuracy of their answers; the values were summed to determine the knowledge scores. Finally, the burden benefit questionnaire recorded the percentage of participants that found the study burdensome or beneficial and why. Additional information about household members and friends helping with medication pickup was also included. The goal was to evaluate the differences between the control group and intervention group. Multiple forms of questions helped determine all of the pharmacies where the participants filled their prescriptions. This information was utilized to determine when and if they filled their DAPT prescriptions.

Upon completion of the surveys, the research participants were randomized using block randomization as part of the clinical trial management system to either the control arm (usual care) or the interventional arm (MyIDEA supplementation). The participant in the interventional arm had the MyIDEA program customized to their PCI procedure results. MyIDEA was a customized educational tablet with patient narratives (short stories about patients with the common reason for DAPT discontinuation) to educate them about the importance of DAPT [21]. Full details of patient participatory design are given elsewhere [21]. The control arm participants were given the opportunity to play checkers or tic-tac-toe to control for the novelty of interacting with a tablet. The control arm had traditional educational material that included commercially prepared material. The control group were not asked questions about the material they received, as the study could have approached them before the formal patient education. The nurse that gave the participant the tablet occasionally helped the participant advance the app but was not involved in the intervention. There were no cointerventions. After interacting with the tablet, all patients had their blood drawn to collect the VerifyNow aspirin reactive units (ARU) and P2Y12 reactive units (PRU) [28]. During the PCI procedure, the patients were administered aspirin and a P2Y12 inhibitor (clopidogrel/ticagrelor/prasugrel). These tests were used to measure platelet suppression resulting from the medication. The medication fill rate was collected for a duration of 3 months to calculate pharmacy days covered (PDC).

All research participants were scheduled for a second encounter with the research team during their follow-up cardiology appointment 1 to 2 weeks later. During this second research encounter, the participants first completed the Morisky 8-item medication adherence questionnaire [29], the PCI knowledge questionnaire, and SF-36. After the questionnaires, they interacted with the tablet. Coronary disease–related outcomes such as repeat coronary intervention or myocardial infarction were determined. During this visit, another blood draw was done for the VerifyNow ARU and PRU. The interventional participants interacted with MyIDEA a second time, and the control participants were encouraged to play games on the tablet.

A third appointment was scheduled at 3 months after the initial hospitalization. The same instruments from the second visit were also completed at that visit. One final blood draw was conducted to collect samples for VerifyNow ARU and PRU. Routine phone calls and letters were sent to the participant to encourage follow-up with the research team. All participants who did not attend their visit received at least three follow-up phone calls to reschedule appointments.

For the participants randomized to the interventional arm, additional data were collected during their usage of the MyIDEA program. While using MyIDEA, participants were asked questions, and their responses were recorded using the tablet microphone. The responses reflected the participants’ learning in their module and the symptoms before the procedure. In addition, time stamps of each click or change in the program were recorded. Five patient stories were weaved throughout the program; participants were asked to record which patient narrative they most related to. No outcomes were changed throughout the study.

### Statistical Analysis

Although the data were extracted and compiled, the research assistants who did not consent the participants and the principal investigator-maintained blinding of the participants. The first 24 participants were unblinded for an intermediate evaluation [27]. However, this study is a complete analysis of all 45 participants. The research team that contacted individuals for recruitment, calculated the PDC, and collected analytics were blinded. However, the nurses that consented participants, the ones who gave the participants the tablet, and the participants were unblinded. The group assignment for all participants was unblinded when the biostatistician calculated the results. The multidisciplinary team and patient advisors reviewed the results and analysis. Descriptive statistics for outcomes and patient characteristics were calculated. A regression analysis of the VerifyNow ARU and PRU was conducted to compare the 2 groups. An imputation of the missing ARU and PRU was conducted via a multivariate imputation by chained by R 3.4.1 (R Foundation)[30]. For other group comparisons, an independent two-tailed *t* test, a Fisher test, a Mann-Whitney test, or a chi-square test was applied.

To examine the correlation between the level of engagement and the time spent responding to questions in the interventional group, recordings from the participants were transcribed by OE and analyzed. Upon completing the transcription, each participant was labeled as engaged or unengaged.

Engagement was determined by using the tone of the participant’s voice and the total length of recording, which could include background noise, nurses, and pauses. More specifically, tone was described as being attentive or disregarding. Participant speaking time was calculated using Adobe Audition, isolating the voice of the participant for the time calculation. The total time the participant spent talking on MyIDEA was calculated for each visit. The average time each participant spent talking on the app was averaged across visits. To test for significance, a Mann-Whitney test was performed using a 0.05 significance level. A secondary analysis looked at engaged vs unengaged and the PCI knowledge questionnaire.

## Results

**Figure 1:**
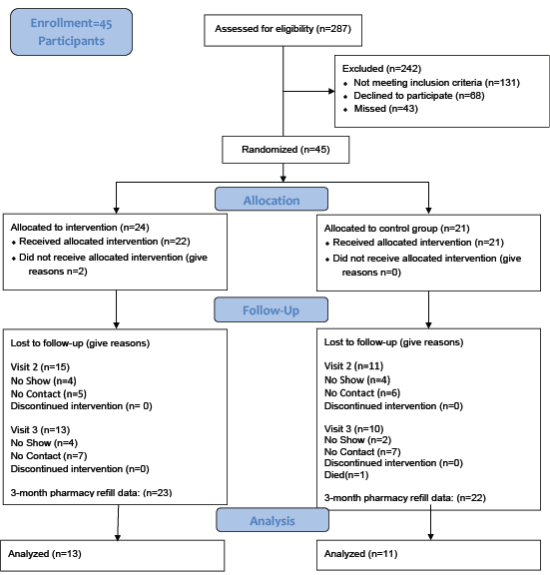
Flow diagram of research participants.

A total of 287 potential research participants were eligible to be recruited during the study at the 2 hospitals (Figure 1). A total of 131 participants did not meet the inclusion criteria, as many of the patients spoke Spanish, and 18 of the potential participants were younger than 50 years. A total of 43 potential participants were missed (ie, the research team was notified but did not approach the participant). For example, 33 potential participants from Cook County had a PCI on the weekend when no research staff was available to recruit.

A total of 45 participants were recruited to the study. One participant withdrew during the first encounter. A total of 23 participants were allocated to the intervention, and 21 participants were allocated to the control. Of the 23 participants randomized to the intervention, 22 participants were given the intervention, as 1 participant left the hospital before the information was provided. In the intervention group, 15 participants attended the second visit, and 13 participants attended the third visit. Of the 21 randomized to the control group, 11 attended the second visit, and 11 attended the third visit. A total of 44 research participants had data from pharmacy refills, as 1 participant withdrew. One participant died in the control group. In 17 blood draws out of the potential 131 blood draws, there was a processing error in both or one of the blood draws. The most common reason was a *processing error* in the lab (11 times; see Figure 1)

The preintervention demographics are outlined in Table 1. The mean age of the participants was 60.4 years (SD 7.1). The mean ages between groups were not significantly different. Of the participants, 60% (27/45) were men, and 40% (18/45) were women (see Table 1). In the control group, 68% (15/24) were men and 32% (7/24) were women, whereas in the MyIDEA group, 48% (11/21) were men and 52% (12/21) were women, but the difference was not statistically significant. The participants were 27% (12/45) white, 60% (27/45) black, 7% (3/45) Asian, and 7% (3/45) multiracial and 7% (3/35) Hispanic. For both groups, the majority of the patients were black, with white being the second largest group, and the rest of the patients being evenly split between Asian and multiple races. The grade reading level of the participants was 2% (1/45) for less than third grade, 9% (4/45) for between fourth and sixth grade, 47% (21/45) for between seventh and eighth grade, and 42% (19/45) for ninth grade and above per REALM-SF. For both the control and MyIDEA groups, the majority of patients’ reading level was seventh grade or above. For medication pickup assistance, 78% of patients (77% of control and 78% of intervention groups) had friends or family who could help pick up prescriptions. The individuals who would assist in medication pickup also benefited from the basic instructions for better conformance to postprocedure instructions from MyIDEA if they were present during the study.

**Table 1:** Preintervention data for all participant, control, and interventional (My Interventional Drug-Eluting Stent Educational App) groups.

Data category			All	Control	MyIDEA^a^	*P* value	
Age (years), mean (SD)			60.4 (7.0)	60.9 (6.9)	60.0 (7.1)	.67	
**Gender, n (%)**							**.28**
	Male		27(60)	15 (68)	11 (48)		
	Female		18 (40)	7 (32)	12 (52)		
**Race, n (%)**							**.88**
	White		12 (27)	6 (27)	6 (26)		
	Black		27 (60)	14 (64)	13 (57)		
	Asian		3 (7)	1 (5)	2 (9)		
	Multiple races		3 (7)	1 (5)	2 (9)		
**Ethnicity, n (%)**							**.53**
	Hispanic		3 (7)	2 (9)	1 (4)		
	Non-Hispanic		42 (93)	20 (91)	22 (96)		
**Medication pickup assistance, n (%)**							
	Patients who had assistance picking up medication		35 (78)	17(77)	18(78)	.94	
**Rapid Estimate of Adult Literacy in Medicine-Short Form n (%)**							**.71**
	<3		1 (2)	0 (0)	1 (4)		
	4-6		4 (9)	3 (14)	1 (4)		
	7-8		21 (47)	11 (50)	10 (43)		
	9+		19 (42)	8 (36)	11 (49)		
**36-Item Short Form Health Survey, value on the test (SD)**							
	Physical functioning		52.7 (30.3)	47.3 (30.3)	57.8 (30.1)	.26	
	Role limitations because of physical health		35.2 (40.5)	30.0 (40.2)	40.2 (41.1)	.41	
	Role limitations because of emotional problems		57.8 (45.0)	56.1 (47.3)	59.4 (43.8)	.81	
	Energy and fatigue		46.0 (19.8)	42.5 (18.8)	49.3 (20.5)	.26	
	Emotional well-being		74.3 (19.3)	70.4 (20.5)	78.1 (17.6)	.19	
	Social functioning		63.3 (29.6)	62.4 (27.6)	64.1 (32.0)	.85	
	Pain		54.0 (29.4)	51.2 (31.8)	56.7 (27.2)	.54	
	General health		52.4 (19.2)	50.7 (18.0)	53.9 (20.5)	.59	
**Hospital Anxiety and Depression Scale, value on the test (SD)**							
	Anxiety		5.8 (4.5)	7.3 (4.5)	4.3 (4.0)	.03	
	Depression		4.5 (3.1)	5.3 (3.3)	3.8 (2.7)	.11	
**Burden-Benefit, n (%)**							
	**Was participation burdensome to you in any way?**						
		Not at all	30 (68)	16 (76)	14 (61)	N/A^b^	
		A little bit	7 (16)	1 (5)	6 (26)	N/A	
		Somewhat	5 (11)	3 (14)	2 (9)	N/A	
		Quite a bit	1 (2)	1 (5)	0 (0)	N/A	
		Very much	0 (0)	0 (0)	0 (0)	N/A	
		No answer	1 (2)	0 (0)	0 (0)	N/A	
	**Was participation beneficial to you in any way?**						
		Not at all	3 (7)	2 (10)	1 (4)	N/A	
		A little bit	6 (14)	2 (10)	4 (17)	N/A	
		Somewhat	16 (36)	10 (48)	6 (26)	N/A	
		Quite a bit	13 (30)	6 (29)	7 (30)	N/A	
		Very much	4 (9)	1 (5)	3 (13)	N/A	
		No answer	2 (4)	0 (0)	2 (9)	N/A	
**Percutaneous coronary intervention** **k** **nowledge**							
	Visit 1		7.9 (1.5)	7.9 (1.5)	7.9 (1.5)	.93	

^a^MyIDEA: My Interventional Drug-Eluting Stent Educational App.

^b^N/A: not applicable.

The HADS anxiety and depression scores for MyIDEA (mean 4.3, SD 4 anxiety and mean 3.8, SD 2.7 depression) and control groups (mean 7.3, SD 4.5 anxiety and mean 5.3, SD 3.3 depression) were statistically significantly different for anxiety. HADS has scores ranging from 0 to 21, where 0 to 7 is normal, 8 to 10 is borderline, and 11 to 21 is abnormal before the intervention. The depression and anxiety were only measured on enrollment. The group differences in average PCI knowledge questionnaire scores or in Morisky medication adherence belief scores for both visits were not statistically significant.

The majority of participants in both the MyIDEA and control groups did not find the study burdensome (65% of MyIDEA and 76% of control groups). However, the two major reasons that the study was burdensome to some patients were that either the interview was too long or there were too many questions and the patient was too weak or ill at times. Similarly, the majority of participants in both the MyIDEA and control groups found participation in the study at least somewhat beneficial. The top two reasons for which they found the study beneficial were that it helped them to think about these topics and that it made them feel good to help others or contribute to society.

SF-36 was given at the first visit, and scores were reported within the normal ranges (see Table 1 and 2).

A comparison of MyIDEA and control groups for medication adherence showed no significant difference in ARU, PRU, or PDC (Tables 3 and 4). The prescription pattern changed midstudy from 30-day prescriptions to 90-day prescriptions. The overall well-being throughout the study was evaluated by using SF-36. The group differences in various domains including energy and fatigue, emotional well-being, social functioning, pain, and general health were not statistically significant. There was a little change from the hospital to the second and third visits.

**Figure 2:**
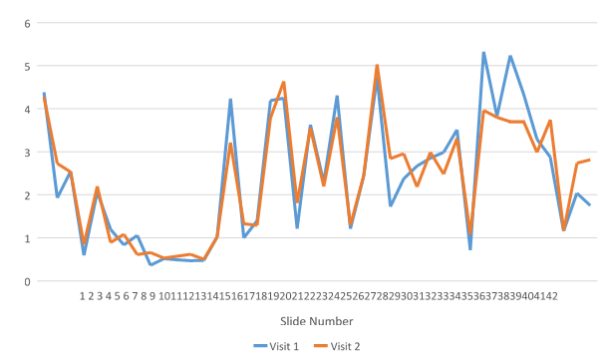
The average percentage of time spent on slides.

For the patients in the MyIDEA group, the average amount of time spent on the app in the first visit was 17.2 min (SD 4.4) and in the second visit was 13.8 min (SD 5.5). The average percentage of time spent on the slides was lowest for the tutorial and chapter introduction slides (Figure 2). The selection of patient narratives is reported in Table 5.

**Table 2:** Postintervention variables.

Postintervention variable				All scoring unit, mean (SD)		Control scoring unit, mean (SD)		Interventional scoring unit, mean (SD)		*P* value
**Visit 2**										
	Morisky			2.4 (0.7)		2.5 (0.6)		2.3 (0.7)		.50
	Percutaneous coronary intervention knowledge			7.9 (1.6)		7.7 (1.5)		8.0 (1.7)		.52
	**36-Item Short Form Health Survey**									
		Physical functioning	55.9 (30.4)		53.5 (30.3)		58.2 (30.2)		N/A^a^	
		Role limitations due to physical health	43.5 (40.0)		38.5 (40.0)		48.2 (39.5)		N/A	
		Role limitations due to emotional problems	60.5 (42.6)		53.8 (40.3)		66.7 (43.6)		N/A	
		Energy and fatigue	54.3 (42.6)		52.3 (22.4)		56.1 (19.4)		N/A	
		Emotional well-being	54.3 (21.0)		70.8 (17.8)		86.9 (12.1)		N/A	
		Social functioning	76.9 (24.0)		72.1 (21.5)		81.3 (25.3)		N/A	
		Pain	66.3 (29.8)		63.7 (28.5)		68.8 (30.8)		N/A	
		General health	58.9 (18.5)		57.7 (19.6)		60.0 (17.3)		N/A	
**Visit 3**										
	Morisky			2.5 (1.4)		2.5 (1.3)		2.5 (1.6)		.94
	**36-Item Short Form Health Survey**									
		Physical functioning	52.0 (27.8)		31.7 (23.3)		68.6 (20.9)		N/A	
		Role limitations due to physical health	45.0 (46.5)		22.2 (44.1)		63.6 (43.8)		N/A	
		Role limitations due to emotional problems	65.0 (44.1)		63.0 (45.5)		66.7 (47.1)		N/A	
		Energy and fatigue	53.8 (18.8)		50.6 (20.2)		56.4 (19.1)		N/A	
		Emotional well-being	75.4 (14.7)		73.8 (15.1)		76.7 (15.6)		N/A	
		Social functioning	75.6 (25.1)		72.2 (29.2)		78.4 (23.8)		N/A	
		Pain	62.0 (28.4)		51.7 (32.2)		70.5 (24.5)		N/A	
		General health	52.0 (12.8)		43.9 (11.7)		58.6 (10.5)		N/A	
	**Proportion of days covered (PDC)**									
		PDC >0.8	N/A		14 (78)		16 (80)		.99	
		PDC ≤0.8	N/A		4 (22)		4 (20)		N/A	
		Adverse events (participants)	N/A		N/A		N/A		N/A	
		Death	N/A		1		0		N/A	
		Rehospitalizations all	N/A		8		11 (9)		N/A	
		Unplanned heart-related hospitalizations	N/A		2		4 (3)		N/A	

^a^N/A: Not applicable.

**Table 3:** Aspirin reactive units and P2Y12 reactive units (PRU) with tablet data. Linear regression of aspirin reactive units (ARU) and PRU.

Dependent variable and predictor		Estimate	SE	*t* value	*P* value
**ARU^a^**					
	Group (reference: control)	10.55	22.56	0.47	.64
	Baseline ARU	0.09	0.16	0.58	.57
**PRU^b^**					
	Group (reference: control)	−9.42	22.39	−0.42	.68
	Baseline PRU	0.52	0.13	3.9	.001

^a^ARU: aspirin reactive units.

^b^PRU: P2Y12 reactive units.

**Table 4:** The average time the research subjects spoke when recording answers to questions sorted by engaged and unengaged participants.

Average time on app	Engaged (seconds)	Unengaged (seconds)
Sample SD	9.35	2.01
Variance	87.41	4.03
Total number	9	13
Sum	111.86	44.29
Mean	12.43	3.41

**Table 5:** Patient selection of most similar to during My Interventional Drug-Eluting Stent Educational App program.

Patient narrative name	First interaction (number of subjects selecting)	Second interaction (number of subjects selecting)
Blank	4	1
Frank	7	1
Eva	10	8
Heather	1	0
Joanne	0	1

A participant who was attentive spent, on average, a longer time on the app than a participant who was disengaged. The length of time a research participant spent on each slide was associated with the tone and content, as determined by the trained listener. It was determined that a participant labeled as engaged spoke for an average of 12.4 seconds per question, whereas an unengaged patient spoke an average of 3.4 seconds per question. The Mann-Whitney test yielded a value *P*<.001. We compared the engaged vs the unengaged for the reading grade level of both groups. A *t* test comparing the scores gives *P*=.09. A Fisher test of proportion of high school graduates between the 2 groups is *P*=.33. The average PCI score for engaged participants was 8.3 and for the unengaged was 7.6.

## Discussion

### Principal Findings

Adherence to DAPT is a key element to optimal prevention of negative outcomes post DES placement. MyIDEA demonstrated the feasibility of elderly patients (>50 years) who were able and willing to learn via a tablet device. There was no significant difference between the groups DAPT and PDC because of the small sample size. However, this test only measures recent medication adherence. In this study, PDC was confounded because of the change in practice from monthly prescriptions to 3-month prescriptions for the P2Y12 inhibitors. This change increased the participants’ PDC.

Elderly patients are willing to use a tablet device to increase their knowledge about medication adherence. Additional studies with larger samples are needed to measure the true clinical impact of novel educational interventions. As technology continues to advance, the challenge of transforming detailed clinical information into a form digestible by patients will continue to need innovation. The difference in time of the respondents between the engaged and the unengaged could be used to encourage health practitioners to identify patients in need of more education or a diversity of education. As this study had a challenge recruiting research subjects, all of the perspectives and potential implications of a hospital-wide deployment of a similar educational program would be challenging to predict. Furthermore, very few subjects found the study burdensome, but the potential benefit was a more nuanced answer with 20% saying “a little bit to not at all” for benefits.

### Intriguing Findings

One surprising finding was the knowledge between the 2 groups was not statistically different in the 2 groups on the second visit from the PCI knowledge questionnaire. The average for the second visit in the control group was 7.7 and the intervention group was 8.0, with the baseline average from the first visit being 7.9. A number of reasons could cause this observation. One is a ceiling effect where you can only score 10 as the highest on the questionnaire. Second, the questionnaire was administered 2 to 4 weeks after the intervention but before the second interaction with the tablet. This was a test of the recall from weeks ago and not immediate recall. In addition, as the MyIDEA educational program focused on both factual information as well as patient narratives about the impact of missing medication, the quiz only focused on factual knowledge. Future knowledge quizzes should focus on applied knowledge as well to measure the complete impact of this type of education.

The MyIDEA and control groups’ SF-36 scores were within the average SF-36 scores for patients with ischemic heart disease (IHD) in the United States [31]. The physical functioning scores for the intervention (57.8) and control (47.3) groups were within the average score of US patients with IHD (58.8, SD 27.4). Similarly, the role limitations due to physical health scores for the intervention (40.2) and control (30) groups were within the range of the average score for US patients with IHD (45.0, SD 42.7). The average energy and fatigue scores of the intervention (49.3) and control (42.4) groups were also within the range of patients with IHD (52.8, SD 20.4).

Emotional well-being scores for the intervention (78.1) and control (71.0) groups were within the range of patients with IHD (73.1, SD 17.9). Social functioning scores for the MyIDEA group (64.1) and the control group (62.5) were also within the range of average scores of patients with IHD (76.0, SD 23.8). The average scores for pain for the interventional (56.7) and control (50.5) groups were also within the average scores for patients with IHD (64.4, SD 25.0). Finally, the average scores for general health for the MyIDEA group (53.9) and the control group (50.5) were within the range for US patients with IHD (54.7, SD 22.2).

### Limitations

The limitations of the study include having only 2 urban hospitals to recruit the research participants. The number of participants recruited was not sufficient for a properly powered study but instead to evaluate feasibility of recruitment and use of the tablet. As tablets and phones have become more interchangeable than when this project first began, a portable version of the program should be designed for future studies. The strength of this study was the ability of the elderly to use a customized tablet program both in the hospital and the outpatient clinic, opening up new possibilities in forms of education.

### Comparison With Commercial Products

Several mobile health apps exist, but these products simply attempt to measure medication adherence. For example, GlowCap reminds patients to take their medication by glowing, sounding an alarm, and calling their homes [32]. However, because the patient only needs to open the medicine bottle to have their adherence recorded, it is not an accurate measurement of adherence. Other apps do not target the reasons behind nonadherence, such as a lack of knowledge about the effects of nonadherence.

MyIDEA was created to intervene before the patient begins to take their medication, whereas other solutions target the patient as they begin their medication. For example, one intervention delivered customized SMS text messages at the time patients were supposed to take their medication for 30 days [32]. This app provides educational reasons to take medicine as well as reminders. However, there is no indication that those patients would always receive the SMS text message or that the patients took the medication. As a result, the educational engagement of this intervention is limited. Another approach to solving adherence is the use of smartphone apps such as MoviPill, a mobile game that connects elderly participants to a social network and awards points for taking their medication close to the prescribed time [33]. This app utilizes social rewards to persuade the patient to take their medication at the right time. However, it only targeted the patients interested in playing the game and did not educate them on why they should take their medicine. Thus, MyIDEA is unique because it begins to educate patients about the importance of medication adherence before they begin to take their medicine and continues the education weeks after the procedure.

As typical with mobile health interventions, the participants were unblinded. Future research could be conducted to compare the level of engagement with medication adherence as well as by working with the clinical team in ensuring patients engage in their own care. If there was a correlation between medication adherence and the level of engagement, MyIDEA could be an effective tool for nurses and other health care providers to better understand the needs of their patients and increase medication adherence.

## Appendix

Multimedia Appendix 1 PCI Knowledge Questionnaire.

Multimedia Appendix 2 CONSORT e-HEALTH checklist (V 1.6.1).

## Abbreviations

ARU: aspirin reactive units
DAPT: dual antiplatelet therapy
DES: drug-eluting stent
HADS: Hospital Anxiety and Depression Scale
IHD: ischemic heart disease
MyIDEA: My Interventional Drug-Eluting Stent Educational App
PCI: percutaneous coronary intervention
PDC: proportion of days covered
PRU: P2Y12 reactive units
REALM-SF: Rapid Estimate of Adult Literacy in Medicine-Short Form
SF-36: 36-Item Short Form Health Survey
