# Supplementary material for: Elderly Medication Adherence Intervention Using the My Interventional Drug-Eluting Stent Educational App: Multisite Randomized Feasibility Trial
Source: JMIR Mhealth Uhealth. 2020 Jun 24;8(6):e15900. doi: 10.2196/15900 (PMC7381043; doi:10.2196/15900)
Supplement: Multimedia Appendix 1 [file mhealth_v8i6e15900_app1.pdf]

Study ID:___________

PCI Questionnaire

What problems lead to developing a heart blockage?

High blood pressure

Diabetes

Smoking

High cholesterol

Are you supposed to take aspirin with *auto-populate with name of drug*?

Yes

No

How long does your doctor want you to take aspirin and *auto-populate with name of drug*?

2 months

6 weeks

1 year

3 months

Why are you taking aspirin and *auto-populate with name of drug*?

Lose weight

Lower cholesterol

Help prevent blood clots in your stent

Help quit smoking

What could possibly happen if you don't take this medication?

Blood pressure will increase

Study ID:________

Stent will clot off and maybe have another heart attack

Cholesterol will go down

Weight will increase

When should you talk to your heart doctor?

If you experience bleeding or bruising

If you are unable to get your medications

If you need to have surgery

If another health care provider asks you to stop these medications

All of the above

How often do you take your *auto-populate with name of drug*?

Every other day

Once a day

Once a week

Monday, Wednesday, Friday
